# Glaucoma Heritability: Molecular Mechanisms of Disease

**DOI:** 10.3390/genes12081135

**Published:** 2021-07-27

**Authors:** Ryan Zukerman, Alon Harris, Francesco Oddone, Brent Siesky, Alice Verticchio Vercellin, Thomas A. Ciulla

**Affiliations:** 1 Department of Ophthalmology, Icahn School of Medicine at Mt. Sinai, New York, NY 10029, USA; ryan.zukerman@gmail.com (R.Z.); alon.harris@mssm.edu (A.H.); brent.siesky@mssm.edu (B.S.); alice.verticchio@mssm.edu (A.V.V.); 2 Department of Ophthalmology, University of Miami Miller School of Medicine, Miami, FL 33136, USA; 3 IRCCS–Fondazione Bietti, 00198 Rome, Italy; oddonef@gmail.com; 4 Midwest Eye Institute, Indianapolis, IN 46290, USA

**Keywords:** glaucoma, genetics, heritability, primary open-angle glaucoma, intraocular pressure, cup-to-disc ratio

## Abstract

Glaucoma is one of the world’s leading causes of irreversible blindness. A complex, multifactorial disease, the underlying pathogenesis and reasons for disease progression are not fully understood. The most common form of glaucoma, primary open-angle glaucoma (POAG), was traditionally understood to be the result of elevated intraocular pressure (IOP), leading to optic nerve damage and functional vision loss. Recently, researchers have suggested that POAG may have an underlying genetic component. In fact, studies of genetic association and heritability have yielded encouraging results showing that glaucoma may be influenced by genetic factors, and estimates for the heritability of POAG and disease-related endophenotypes show encouraging results. However, the vast majority of the underlying genetic variants and their molecular mechanisms have not been elucidated. Several genes have been suggested to have molecular mechanisms contributing to alterations in key endophenotypes such as IOP (*LMX1B*, *MADD*, *NR1H3*, and *SEPT9)*, and VCDR (*ABCA1*, *ELN*, *ASAP1*, and *ATOH7*). Still, genetic studies about glaucoma and its molecular mechanisms are limited by the multifactorial nature of the disease and the large number of genes that have been identified to have an association with glaucoma. Therefore, further study into the molecular mechanisms of the disease itself are required for the future development of therapies targeted at genes leading to POAG endophenotypes and, therefore, increased risk of disease.

## 1. Introduction

Glaucoma, a leading cause of irreversible blindness globally, is a degenerative optic neuropathy characterized by progressive visual field defects corresponding to retinal ganglion cell and retinal nerve fiber layer (RNFL) degeneration [1,2]. Primary open-angle glaucoma (POAG) represents the most common form of glaucoma worldwide, responsible for nearly three-quarters of all cases of glaucoma [1]. POAG is a form of disease characterized by an anatomically open angle; in contrast to primary angle-closure glaucoma (PACG) where anatomic changes obstruct aqueous humor outflow from the anterior chamber, in POAG, drainage of aqueous humor out of the anterior chamber is obstructed despite an anatomy that should allow for aqueous drainage [3]. An elevated intraocular pressure (IOP) represents the major and only currently approved modifiable risk factor for glaucomatous disease onset and disease progression [4]. However, not all patients with glaucoma have elevated IOP, and not all patients with elevated IOP develop glaucoma. Therefore, the pathogenesis of glaucoma is still not fully understood and likely differs among persons and populations.

As researchers have tried to explore and better understand the multifactorial nature of glaucoma, many studies have sought a genetic explanation for the disease. Genetic and genomic studies of single-gene variants, whole-exome sequencing, genome-wide association studies (GWAS), and genetic/polygenic risk scoring have all been used to describe genetic contributions to glaucoma and to evaluate the role of genetics as a risk factor for disease [5]. In addition to these studies, scientists have tried to evaluate the heritability of glaucoma.

Heritability, represented by h^2^, is a complex statistical measurement ranging from zero to one that describes what percent of variability in a trait is due to genetic factors versus environmental factors [6]. For example, characteristics such as language and political ideology have a heritability close to zero—indicating that these characteristics are almost entirely influenced by environmental factors with no genetic influence. Meanwhile, traits caused by single-gene mutations, such as phenylketonuria, have a heritability close to one, indicating that variability in this trait is almost entirely the result of genetic influence [7]. Glaucoma represents a complex, multifactorial trait with a quantifiable genetic influence, and therefore h^2^ estimates for glaucoma fall between zero and one [8].

In 2019, Asefa et al. conducted a systematic review and meta-analysis of glaucoma heritability, and the heritability of glaucoma-related endophenotypes. For glaucoma specifically, their search yielded six studies—three specifically for POAG, two for unspecified glaucoma, and one for PACG—and their pooled h^2^ for POAG ranged from 0.17 to 0.81 [8]. Meanwhile, their pooled h^2^ estimates for POAG endophenotypes included h^2^ estimates for IOP (h^2^ = 0.43), anterior chamber size (h^2^ = 0.67), central corneal thickness (CCT) (h^2^ = 0.81), cup-to-disc ratio (CDR) (h^2^ = 0.56), disc size (h^2^ = 0.61), cup size (h^2^ = 0.58), corneal hysteresis (h^2^ = 0.40), RNFL thickness (h^2^ = 0.73), cup shape (h^2^ = 0.62), and peripapillary atrophy (h^2^ = 0.73) [8].

Since glaucoma is a multifactorial disease, heritability estimates of specific glaucoma endophenotypes and risk factors may be more important clues for researchers as to which endophenotypes, and in turn molecular mechanisms, are important to the pathogenesis and progression of disease. While these estimates for glaucoma and endophenotype heritability are generally strong, the majority of genetic variants underlying these traits are still unknown. Additionally, the molecular etiology of these endophenotypes, and their role in the pathogenesis of glaucoma, is similarly unclear. For instance, it has been suggested that IOP and vertical CDR (VCDR) may represent the true POAG endophenotypes, while CCT may not be physiologically significant, but the molecular mechanisms underlying genetic contributions to these traits are not well understood [9,10].

Therefore, this review aims to summarize the available literature surrounding the heritability of POAG endophenotypes IOP and VCDR, with an emphasis on recently uncovered genes and proposed molecular mechanisms for disease pathogenesis and progression.

## 2. Materials and Methods

PubMed, Embase, Ovid, Scopus, and Trip searches were conducted through 31 May 2021 to evaluate all pertinent articles, abstracts, and ongoing research projects. Searched key words include glaucoma, genetics, genome-wide association study, heritability, primary open-angle glaucoma, genes, intraocular pressure, vertical cup-to-disc ratio, cup-to-disc ratio, and endophenotypes. The same key words were used in all search software. Articles were screened for relevance and analyzed based on inclusion criteria, population, and specific genes studied. Data were collected and organized using Microsoft Word (version 16.30, Microsoft, Microsoft Corporation, Redmond, WA, USA), Microsoft Excel (version 16.30, Microsoft, Microsoft Corporation, Redmond, WA, USA), and EndNote (X8.2, Clarivate Analytics, Clarivate, Philadelphia, PA, USA).

References from all relevant articles found were reviewed to ensure inclusion of all relevant articles.

## 3. Discussion

### 3.1. Intraocular Pressure

Elevated intraocular pressure represents the only currently approved modifiable risk factor for POAG. While the exact molecular mechanisms underlying elevated IOP and its relationship to glaucoma are not fully understood, it is well known that elevated IOP is strongly associated with both structural and functional glaucomatous change [3,11]. Currently, this relationship is described by mechanical strain that elevated IOP places on the lamina cribrosa and adjacent optic nerve tissues, ultimately resulting in structural damage that disrupts axonal transport [3,12]. It has also been suggested that there may be a pressure-induced metabolic stress effect, and even circulatory, immune, and oxidative stress mechanisms associated with glaucoma and related to IOP [3,13,14].

Interestingly, elevated IOP has shown a relatively strong heritability estimate. When Asefa et al. conducted a systematic review and meta-analysis for the heritability of glaucoma, they also evaluated pooled h^2^ estimates for endophenotypes, including IOP, which they estimated at h^2^ = 0.43 (95% confidence interval (CI) 0.38–0.48). Importantly, in their analysis, IOP was the most commonly reported endophenotype, reported in 41 different studies. This large sample also allowed for subgroup analysis, showing that there was heterogeneity between age and ethnicity. Findings showing different heritability estimates between White Europeans (h^2^ = 0.47, 95% CI 0.37–0.57, *p* = 0.006), East Asians (h^2^ = 0.49, 95% CI 0.40–0.59), and Other Populations (h^2^ = 0.30, 95% CI 0.22–0.39) are particularly interesting given suspected genetic contributions to disease and genetic differences among different racial groups [5,8]. Additionally, it should be noted that IOP has also shown a genetic correlation with POAG (odds ratio 1.18, 95% CI 1.14–1.21, *p* = 1.8 × 10^−27^) [15].

To date, a wide variety of genes have been associated with elevated IOP, including, but not limited to: *ABCA1*, *ABO*, *ADAMTS8*, *ADAMTS17*, *ADAMTS18-NUDT7*, *AFAP1*, *ANGPT1*, *ANTXR1*, *ARHGEF12*, *ARID5B*, *ATXN2*, *CAV1-CAV2*, *CDKN2B-AS1*, *CELF1*, *CYP26A1-MYOF*, *FAM125B*, *FNDC3B*, *FOXC1*, *FOXP1*, *GAS7*, *GLCCI1-ICA1*, *GLIS3*, *GMDS*, *HIVEP3*, *INCA1*, *LMX1B*, *LOC171391*, *MADD*, *MIR548F3*, *MYBPC3*, *NDUFS3*, *NR1H3*, *PDDC1*, *PKHD1*, *PTPRJ*, *RAPSN*, *RPLP2-PNPLA2*, *SIX1/SIX6*, *SEPT9*, *SP11*, *TFEC-TES*, *TMCO1*, and *TXNRD2* [5,16]. It should be noted that all of these genes have not also been independently associated with POAG. Similarly, molecular mechanisms of these genes are generally not well understood (Table 1).

In 2018, Gao et al. conducted the largest known GWAS for IOP using patients of European Descent (ED) from the UK Biobank (*n* = 115,486). They identified 671 significant genetic variants associated with IOP, mapping to 149 loci, significantly expanding the known literature and replicating findings from previous studies. Importantly, they specifically identified four significant novel genes: *LMX1B*, *MADD*, *NR1H3*, and *SEPT9* [16].

**Figure 1:**
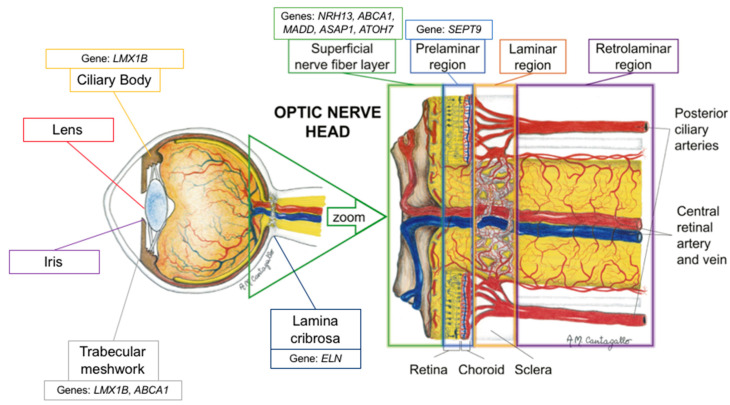
Schematic representation of the proposed location of the genes’ mechanism of action (adapted with permission from: Prada D, Harris A, Guidoboni G, Siesky B, Huang AM, Arciero J. Autoregulation and neurovascular coupling in the optic nerve head. Survey of Ophthalmology 1 March 2016; 61(2): 164–186).

*LMX1B* is a gene that is directly implicated in the development of serotonergic (5-HT) neurons [21]. According to Ding et al., *LMX1B* is a LIM homeodomain-containing gene, which they suggest is a key intermediate factor necessary for the cascade of specification and differentiation of 5-HT neurons [21]. In mouse models, the LMX1B transcription factor has been associated with the regulation of anterior segment development, with *LMX1B* transcripts being identified in the ciliary body and trabecular meshwork (Table 2) [17]. In fact, Pressman et al. described that mice with targeted *LMX1B* mutations have structural deficiencies in the ciliary body, corneal stroma, and iris [17]. If *LMX1B* mutations are associated with alterations in the ciliary body and anterior chamber structures, it is logical to consider that aqueous humor dynamics may be altered, leading to elevated IOP and, potentially, POAG, though further evaluation is surely warranted (Figure 1).

It should be noted that *LMX1B* mutations have been associated with families affected by nail–patella syndrome (NPS), a rare autosomal dominant developmental disorder with variable presentation, and POAG, suggesting an explanation for the association between NPS and glaucoma (approximately 1/3 of patients with NPS develop glaucoma), though not explaining a mechanism for causation [34,35,36]. In 2018, Choquet et al. suggested that the variability in glaucoma phenotypes associated with *LMX1B* may be explained by different mutations. By inducing different *LMX1B* mutations in a murine model, they found that different *LMX1B* mutations can result in elevated IOP and glaucomatous damage independent of developmental defects, suggesting that *LMX1B* is a significant glaucoma-susceptibility locus even in the absence of developmental abnormalities [37]. Still, further research is necessary to better understand the role of *LMX1B* in glaucoma pathogenesis.

*NR1H3* is a nuclear receptor also referred to as the liver X receptor α. Liver X receptors regulate lipid homeostasis, with expression in both the liver and the brain, and play a key role neurodegenerative disease [29]. According to Yang et al., liver X receptor α may also play an anti-inflammatory role, as demonstrated by a mouse model of autoimmune uveitis (Table 2) [30]. In fact, they suggested that liver X receptor α may inhibit the NF-kB signaling pathway, resulting in the reduction in ocular inflammation. These findings reflect those of Zheng at al., which suggest that the activation of liver X receptor β induces a protective effect on the retina perhaps by decreasing retinal amyloid β formation and by inhibiting the NF-kB signaling pathway [31]. While Yang et al. investigated this anti-inflammatory effect in the context of autoimmune uveitis therapeutics, recent research has demonstrated the utility of the anti-inflammatory effect of liver X receptors in relation to glaucoma.

In 2019, Song et al. demonstrated that liver X receptors α and β were expressed in the retina and optic nerve (Figure 1) [32]. They also demonstrated that there was an accumulation of amyloid deposition in the retinal ganglion cells of liver X receptor β knockout mice, and that there was activation of microglia and loss of aquaporin 4 (a biomarker of optic nerve function) in the optic nerve. Ultimately, they reported that the loss of liver X receptor β results in retinal ganglion cell loss and optic nerve degeneration (Table 2) [32]. Interestingly, they noted that aquaporin 4 expression decreased without the presence of aquaporin 4 antibodies, suggesting that optic nerve degeneration in this model is caused by the loss of aquaporin 4 expression [32]. While these findings are encouraging, they do not directly explain the association between *NR1H3* and IOP as suggested by the 2018 GWAS from Gao et al. [16].

The association between *NR1H3* and IOP may actually be explained by *ABCA1*, a POAG-susceptibility locus associated with ED populations as well as Asian populations [5,38,39]. ABCA1, the ATP-binding cassette transporter A1, is a transmembrane transporter involved in cholesterol homeostasis through the formation of high-density lipoprotein [40]. Importantly, ABCA1 is regulated by the liver X receptor [41]. While ABCA1 has been implicated in retinal inflammation and retinal ganglion cell apoptosis, recent research has also demonstrated that ABCA1 has a role in regulating IOP through aqueous humor dynamics [19,22]. In a 2020 study, Hu et al. proposed a pathophysiologic mechanism for the role of ABCA1 in regulating IOP, and an alternate function to its role in cholesterol homeostasis. They suggested that ABCA1 changes Cav1 expression, resulting in a decreased inhibitory influence of Cav1 on eNos, resulting in the increased nitric oxide production by eNOS and, ultimately, reduced IOP (Table 2) [19]. Additionally, they confirmed that ABCA1 had higher expression in the trabecular meshwork of POAG patients versus controls, confirming previous work suggesting the enhanced expression of ABCA1 as a diagnostic marker for glaucoma [19,23]. Therefore, it is possible that variants in *ABCA1* and *NR1H3* result in altered IOP and aqueous outflow regulation.

The third most significant novel gene identified by Gao et al. in their 2018 GWAS was *MADD*, a gene from the MAP kinase activating death domain [16]. *MADD* plays a role in the tumor necrosis factor α (TNF-a) signaling pathway and, therefore, apoptotic signal transduction, by way of mitogen-activated protein kinase (MAPK) [28]. *MADD* has been found to be upregulated in the retinas of POAG patients (Figure 1) [28,42]. This finding reflects those of increased TNF-a and TNF-a receptor 1 in glaucomatous optic nerve heads as measured by immunohistochemistry, suggesting a role for this signaling pathway in the neurodegenerative pathophysiology for POAG [42,43,44].

Currently, there are no studies specifically linking *MADD* expression and IOP. However, TNF-a and TNF-a receptor expression increased by nearly eightfold has been demonstrated in rats with elevated IOP compared to other animal models [45]. Additionally, it has been suggested that TNF-a may indirectly mediate the cytotoxicity of elevated IOP via-microglial activation, and that stimulated glial cells may secrete TNF-a and nitric oxide to initially attenuate the effects of elevated IOP—despite the detrimental downstream effects of microglial activation such as the loss of aquaporin 4 [18,46,47,48]. Therefore, there may be an association between *MADD* expression and IOP, as described by Gao et al. [16]. Still, a 2010 analysis of TNF-a concentrations in the aqueous humor of glaucoma patients found a significant association between TNF-a concentrations and glaucoma patients (mean concentration 15.9 ± 3.6 pg/mL, range 1.7–57.6 pg/mL) but was unable to find a relationship between IOP and TNF-a concentration (*r* = 0.163) [49]. Therefore, the relationship between *MADD*, and by extension TNF-a, and IOP remains unclear.

The final significant novel gene identified by Gao et al. was *SEPT9*, a septin protein [16]. Septins are a group of GTP-binding proteins that function in cell division and cytoskeleton formation; they have also been associated with neurodegenerative diseases [50]. In 2013, Ghossoub et al. described the role of septins in retinal pigment epithelial cells, noting how SEPT2 forms a complex with SEPT7 and SEPT9, with the complex controlling ciliogenesis and, more specifically, the elongation of the primary cilium in cells of the retinal pigment epithelium (Table 2) [33].

The molecular mechanism linking septin activity and intraocular pressure has not been elucidated. It should be noted, though, that microtubule-associated protein 4 appears to play an antagonistic role with the activity of the SEPT2/SEPT7/SEPT9 complex in retinal pigment epithelial cells (Figure 1) [33]. Although not the same protein, one previous immunohistochemical study have shown that acutely increased IOP leads to a decrease in microtubule-associated protein 1 expression and a resulting decrease in the number of microtubules in optic nerve tissues, suggesting a possible mechanism by which cytoskeletal structures may be linked to IOP changes [20].

While these four genes were identified as the most significant by Gao et al. in their 2018 GWAS, it is important to note that these genes are not conclusively the most important genetic markers of IOP. In fact, when Gao et al. calculated a heritability estimate for the UK Biobank sample limited to only significant genetic variants (*n* = 671), the significant variants only explained 7.2% of variance [16]. Additionally, when evaluated for pleiotropy, the 671 significant genetic variants also directly matched SNPs in the GWAS Catalog associated with PACG; age-related macular degeneration; other ocular characteristics such as central corneal thickness, axial length, and iris characteristics; and other systemic traits and conditions such as blood pressure, body mass index, type 2 diabetes mellitus, cancers, and immune disorders [16].

Additionally, it is important to note, that IOP is not a perfect indicator for glaucoma, and therefore it cannot fully explain the heritability, nor the underlying molecular mechanisms, of all cases of POAG. In fact, a large subset of POAG patients do not have elevated IOP. These patients are referred to as having normal-tension glaucoma (NTG)—a unique classification of glaucoma where glaucomatous damage occurs independent of elevated IOP [51]. In fact, Park et al. even suggested that the alteration of LMX1B transcription factor function may lead to glaucomatous damage independent of IOP alterations, as they noted that single-nucleotide polymorphisms (SNPs) of *LMX1B* were also associated with NTG—and highlighting the danger of defining and evaluating POAG singularly through a lens of elevated IOP [36].

### 3.2. Vertical Cup-to-Disc Ratio

In addition to IOP, CDR has been similarly suggested to be a “true” POAG endophenotype [9]. VCDR is a reflection of the loss of retinal ganglion cells and axons, which results in the thinning of the neuroretinal rim and is represented clinically by alterations to the VCDR [52]. Similar to IOP, VCDR is an imperfect indicator of disease. According to Tatham et al., VCDR has limited ability to detect glaucoma given the wide variability of VCDR in healthy eyes [53]. Additionally, even though VCDR reflects retinal ganglion cell loss, VCDR is not a strong method for estimating quantitative measurements of retinal ganglion cell loss, largely due to wide variability of optic nerve head parameters [53]. In fact, in a large population-based study of healthy eyes (*n* = 6616), Hopley et al. found that the wide variability in normal CDR is influenced at least in part by the size of the optic disc [54]. Still, according to a large systematic review from Hollands et al., increased CDR is a key risk factor for POAG, as the likelihood for POAG increases as CDR increases. [55].

In their 2019 systematic review and meta-analysis, Asefa et al. calculated a heritability estimate for CDR at h^2^ = 0.56 (0.44–0.68) [8]. Importantly, they suggested that the wide variability in heritability estimates of CDR may be a reflection of the variability in cup size in the general population. Still, CDR shows relatively high heritability as a trait.

Genes significantly associated with CDR include, but are not limited to: *ABCA1*, *ABG*, *ADAMTS8*, *ASAP1*, *ASB7*, *ATOH7*, *ATOH7-PBLD*, *BMP2*, *CARD10*, *CDC7-TGFBR3*, *CDKN2B*, *CDKN2B-CDKN2BAS*, *CHEK2*, *COL8A1*, *CRISPLD1*, *DCLK1*, *DGKB*, *DUSP1*, *ELN*, *ENO4*, *EXOC2*, *F5*, *FAM101A*, *GAS7*, *HSF2*, *PDZD2*, *PLCE1*, *PSCA*, *RARB*, *RERE*, *RPAP3*, *RPE65*, *RREB1*, *SALL1*, *SCYL1*, *SIX1*, *SIX6*, *SSSCA1*, *TMTC2*, and *VCAN* [56,57,58,59,60,61,62]. Several of these genes, including *ABCA1*, *ABG*, *AFAP1*, *CAV1*, *GAS7*, and *LMX1B* have been independently associated with both IOP and CDR [59,61]. Importantly, however, not all of these genes have also been independently associated with POAG.

In their 2017 GWAS and meta-analysis, Springelkamp et al. identified a variety of novel genes influencing CDR and other POAG endophenotypes. In order to better understand the molecular mechanisms behind these genes, they conducted a pathway analysis using DEPICT, a genetic association tool intended to predict gene function [63]. Their findings were diverse; for example, they noted that genes associated with CDR, cup area and disc area were associated with biologic pathways associated with metabolic processes [59]. Given redundancy between significant pathways, they clustered pathways into a series of meta-pathways, noting that gene sets centered on pathways involving cell differentiation and other developmental pathways [59].

Interestingly, the meta-pathways Springelkamp et al. developed singled out pathways associated with abnormal fat cell and liver morphologies, noting that these pathways centered on *ABCA1* [59]. As described above, *ABCA1* is a transmembrane transporter with a role in cholesterol processing that has recently been linked to IOP regulation and aqueous humor dynamics (Table 3) [19]. So, while this recent development explains the linkage between *ABCA1*, IOP, and the meta-pathways described by Springelkamp et al., the link to CDR is still unclear.

In 2014, Chen et al. found that *ABCA1* was highly expressed in the retinal ganglion cell layer and is significantly higher in POAG patients. They suggested that this high expression indicates that *ABCA1* plays a role in the normal functioning and cell death of retinal ganglion cells [64]. Given that CDR is a reflection of retinal ganglion cell death, it is likely that, in addition to its suggested role in regulating IOP and aqueous humor dynamics, *ABCA1* is linked to CDR through a molecular mechanism involving retinal ganglion cell death (Figure 1). It is important to note that in 2018 Li et al. demonstrated that the liver X receptor/ABCA1 pathway may actually be protective against retinal ganglion cell apoptosis (Table 2) [22]. Their model, however, noted reduced ABCA1 retinal expression in response to acute IOP elevation, which may not be reflective of the role of ABCA1 protein in POAG and its link to CDR. Additionally, it is possible that associations between CDR and *ABCA1* may be reflective of IOP-mediated retinal ganglion cell death. Therefore, further research is surely warranted to better understand the true mechanistic relationship between these closely intertwined variables.

In 2021, Han et al. conducted the largest known GWAS for VCDR and vertical disc diameter (VDD) using by utilizing artificial intelligence (AI) in the form of a convolutional neural network model. Using this technology they were able to analyze VCDR and VDD from 282,100 images from the UK Biobank and another independent study and then were able to systematically use these studies to conduct a stronger GWAS of optic nerve head parameters [61]. Using this data, they compared heritability estimates for VCDR as calculated from clinician gradings of CDR versus calculated from the AI-based GWAS. They found that the SNP-based heritability estimates were nearly 50% higher (VCDR h^2^ = 0.22 vs. 0.35), indicating a future role for AI in heritability studies. In fact, they suggested that AI techniques may lead to higher heritability estimates in twin-studies [61].

In addition to stronger heritability studies, the AI-assisted GWAS more than doubled the number of identified genetic loci for VCDR and VDD, similarly uncovering new mechanistic pathways. For example, Han et al. described new VCDR genes with possible links to retinal ganglion cell biology. Among these, a missense variant in the elastin gene, *ELN*, was particularly noteworthy [61].

Elastin is an extracellular matrix protein that has a significant role in tissue strength and elasticity [67]. In the eye, elastin has been found extensively throughout the choroid, conjunctiva, meninges, muscle tendons and, most densely, in the sclera surrounding the optic nerve head (Figure 1) [26]. Given its function, elastin is a key component to normal ocular physiology as the sclera must be able to withstand the constant mechanical strain and stress induced by normal IOP and ocular blood flow pulsations (Table 2) [26]. Therefore, an alteration to the function of elastin and its ability to permit elastic structural movement and recovery, could have a dramatic degenerative effect. In the case of *ELN* and VCDR, it is logical that alterations to the connective tissue of the lamina cribrosa could predispose a patient to glaucomatous damage and, as a result, increased VCDR.

Interestingly, in 2021, Alipanahi et al. ran a similar study using AI to enhance the power of a GWAS. Using a machine learning model based on 81,830 ophthalmologist-labeled fundus images to phenotype VCDR, they predicted VCDR on 65,680 participants from the UK Biobank. Then, using the machine learning-generated VCDR, they performed a GWAS to evaluate for genetic associations with VCDR. They uncovered 93 novel loci associated with VCDR [65].

Among the novel genetic loci they uncovered was a SNP near *ASAP1*, a locus previously associated with glioma [65,68]. Recent research has demonstrated that glaucomatous retinal ganglion cell loss, and therefore clinical measurements of VCDR, may in some cases be mediated by glial cells [66]. For example, the interplay of microglial activation and nitric oxide, as described previously, can be a beneficial response to elevations in IOP but may also lead to the downstream loss of aquaporin channels, leading to optic nerve degeneration [46]. In the case of *ASAP1*, Alipanahi et al. suggested that this association may indicate a mechanism by which glial cells mediate VCDR (Table 2) [65].

An important example of a glial cell-mediated mechanism that would lead to glaucomatous damage reflected clinically by VCDR is the gene *ATOH7*, which has been significantly associated with VCDR [56,57,69]. The ATHO7 protein, Atonal BHLH Transcription Factor 7, is a key transcription factor for the differentiation of Müller cells, the key retinal glial cell [24,70]. According to Song et al., ATOH7 may promote the differentiation of retinal Müller cell-derived stem cells into retinal ganglion cells (Table 2) [24]. In fact, recent studies have shown that ATOH7 is a critical regulator of retinal ganglion cell genesis (Figure 1). In 2020, Miesfeld et al. demonstrated that retinal ganglion cell genesis was strictly dependent on *ATOH7* expression (Table 2) [25]. Using a mouse model, they demonstrated how the number of adult retinal ganglion cells was directly correlated with *ATOH7* expression. Additionally, they noted how *ATOH7* mutants with decreased retinal ganglion cells experienced a variety of downstream effects and secondary malformations, including alterations to the retinal vasculature and optic nerve head parameters [25]. These findings demonstrate that the association between *ATOH7* and VCDR may be mediated by the effect of the ATOH7 protein on glial cell differentiation into retinal ganglion cells, indicating one mechanism by which retinal glial cell alterations secondary to genetic alterations may predispose patients to alterations in VCDR.

## 4. Conclusions and Future Directions

While genetic and genomic studies have demonstrated the heritability of POAG endophenotypes such as IOP and VCDR, it is important to stress the polygenic multifactorial nature of POAG as a whole. POAG is a complex disease with an unclear mechanism and even less clear inheritance pattern. Take, for example, the two POAG endophenotypes discussed in this review—IOP and VCDR. While these traits are considered the “true” POAG endophenotypes [9], these traits do not exist in a vacuum and likely contribute to each other in some mechanistic fashion, i.e., through IOP-mediated retinal ganglion cell degeneration, leading to increased VCDR.

Alipanahi et al. described this challenge in their AI-enhanced GWAS. They suggested that genetic variation in VCDR may be mediated by pathophysiologic processes involving IOP and the anterior segment of the eye. In fact, they found that there was a 13% overlap between genes in an IOP GWASA meta-analysis and their new VCDR GWAS [65]. Similarly, proposed molecular mechanisms linking genes to IOP and/or VCDR may have similar pathways, whether through glial activation or inactivation or through alterations in aqueous humor dynamics.

Additionally, it should be noted that other endophenotypes associated with POAG have similar heritability estimates. According to Asefa et al., pooled h^2^ estimates for POAG endophenotypes included h^2^ estimates for IOP (h^2^ = 0.43), anterior chamber size (h^2^ = 0.67), central corneal thickness (CCT) (h^2^ = 0.81), cup-to-disc ratio (CDR) (h^2^ = 0.56), disc size (h^2^ = 0.61), cup size (h^2^ = 0.58), corneal hysteresis (h^2^ = 0.40), RNFL thickness (h^2^ = 0.73), cup shape (h^2^ = 0.62), and peripapillary atrophy (h^2^ = 0.73) [8]. In addition to the challenge associated with the sheer number of genes currently associated with POAG, IOP, and VCDR, there are considerably more genes and potential molecular mechanisms still to be uncovered for each of these traits both individually and together.

Still, endophenotypes are a key method for the evaluation glaucoma heritability and may help with eventual individualized medicine practice for the clinical management of glaucoma. For example, the AI-enhanced GWAS performed by Alipanahi et al. was able to use the AI-enhanced models to calculate genetic/polygenic risk scores (GRS/PRS), a method of quantifying risk based on individualized genetic characteristics. Alipanahi et al. calculated a PRS for VCDR that they proposed could be applied to POAG, and they demonstrated that patients with a higher VCDR PRS had a higher prevalence of POAG [65].

An important component of individualized medicine practices is recognition and understanding of the racial disparities associated with disease and outcomes. Specifically, POAG has been shown to present earlier and be more severe in patients of African descent as compared to those of European descent, and populations of African descent have a considerably higher disease burden as demonstrated by differences in disease prevalence across different populations [2,5]. Similarly, PACG is more prevalent in Asian populations as compared to European and African populations [2,5], while the mechanisms behind differences in risk remain largely unconfirmed. Further research into genetic differences among people of different races may further the understanding of glaucoma risk factors across populations and, ultimately, help achieve individualized medicine practices to improve outcomes and reduce disease disparities.

Aside from individualized medicine practices, better understanding of POAG and endophenotype molecular mechanisms has the potential to dramatically reshape the management of glaucoma at the population-level through the development of targeted therapeutics. As more genes and molecular pathways are uncovered, therapies aimed at specific molecular mechanisms may be developed and tested. For example, in 2020, Wu et al. described an application of a gene therapy injectable to disrupt the aquaporin 1 locus in the ciliary body to reduce aqueous humor production and, therefore, reduce IOP to prevent retinal ganglion cell loss [71,72]. Similarly, it has been suggested that gene therapy may be used for retinal ganglion cell neuroprotection [73]. However, to this point, therapies targeted specifically at genes leading to POAG endophenotypes and, therefore, increased risk of POAG have not been identified. In order to specifically target these genes, it is necessary to develop a better understanding of their exact role in causing POAG endophenotypes and disease.

Therefore, it is important that work continues to uncover the molecular mechanisms of disease. Given the large number of genes that have been uncovered, this presents a considerable challenge. One method to uncover these molecular mechanisms and provide more guided avenues for study may be through the use of gene set enrichment as was performed by Springelkamp et al. using DEPICT [59,63]. In fact, the augmentation of AI-enhanced datasets and GWAS with such analytic programs presents an exciting future avenue of research.

Ultimately, in order for genetic and genomic analysis to play a large role in the management of glaucoma, it will be important to better understand the molecular mechanisms that lead to POAG endophenotypes and disease itself. By better understanding the genetics of glaucoma and heritability, as well as the underlying genetic mechanisms, we will be able to expand glaucoma screening, diagnosis, and management systems to better serve our patients impacted by disease.

## Figures and Tables

**Table 1:** Selection of genes associated with IOP and suggested molecular mechanisms.

Gene	GWAS Identified	Suggested Mechanism	Reference
*LMX1B*	Gao et al., 2018 [16]	Altered Anterior Segment Development; Altered Aqueous Humor Dynamics	Pressman et al., 2000 [17]
*MADD*	Gao et al., 2018 [16]	TNF-a-Mediated Microglial Activation	Tezel et al., 2000 [18]
*NR1H3*	Gao et al., 2018 [16]	Alteration of IOP via-ABCA1-Regulated Aqueous Humor Dynamic Alterations	Hu et al., 2020 [19]
*SEPT9*	Gao et al., 2018 [16]	Cytoskeletal Alterations	Ou et al., 1998 [20]

**Table 2:** List of IOP and POAG genes under investigation.

Gene	Study	Environment
ABCA1	Hu et al., 2020 [19]	Ex vivo Human Model
ABCA1	Li et al., 2018 [22]	In vivo Mouse Model
ABCA1	Yeghiazaryan et al., 2005 [23]	In vivo Human Model
ATOH7	Song et al., 2015 [24]	In vivo Rat Model
ATOH7	Miesfeld et al., 2020 [25]	In vivo Mouse Model
ELN	Gelman et al., 2010 [26]	Ex vivo Mouse Model
LMX1B	Cross et al., 2014 [27]	In vivo Mouse Model
LMX1B	Pressman et al., 2000 [17]	In vivo Mouse Model
MADD	Schievella et al., 1997 [28]	In vitro Yeast Interaction Trap
NR1H3	Wang et al., 2002 [29]	In vivo Mouse Model
NR1H3	Yang et al., 2014 [30]	In vivo Mouse Model
NR1H3	Zheng et al., 2015 [31]	In vivo Mouse Model
NR1H3	Song et al., 2019 [32]	Ex vivo Mouse Model
SEPT9	Ghossoub et al., 2013 [33]	Ex vivo Human Model
SEPT9	Ou et al., 1998 [20]	In vivo Guinea Pig Model

**Table 3:** Selection of genes associated with CDR and suggested molecular mechanisms.

Gene	GWAS Identified	Suggested Mechanism	Reference
*ABCA1*	Springelkamp et al., 2017 [59]	Normal Function and Cell Death of Retinal Ganglion Cells	Chen et al., 2014 [64]
*ELN*	Han et al., 2021 [61]	Alteration to Normal Function of Elastin, Leading to Optic Nerve Head Degeneration	Gelman et al., 2010 [26]
*ASAP1*	Alipanahi et al., 2021 [65]	Glial Cell-Mediated Retinal Ganglion Cell Loss	García-Bermúdez et al., 2021 [66]
*ATOH7*	Nannini et al., 2018 [60]	Alteration to Müller Cel Differentiation and Retinal Ganglion Cell Genesis	Miesfeld et al., 2020 [25]

## Data Availability

No new data were created or analyzed in this study. Data sharing is not applicable to this article.
